# Exoscope versus microscope in spine surgery: A meta-analysis based on procedure-specific subgroup analysis

**DOI:** 10.1007/s10143-026-04275-0

**Published:** 2026-04-10

**Authors:** Alberto Benato, Donato Creatura, Davide Palombi, Cédric Yves Barrey

**Affiliations:** 1 Department of Spine and Spinal Cord Surgery, Hospices Civils de Lyon, Lyon, France; 2 Department of Neurosurgery, Humanitas Research Hospital, Milan, Italy; 3 Department of Pediatric Neurosurgery, Fondazione Policlinico “A. Gemelli”, Rome, Italy; 4 University Claude Bernard of Lyon 1, Lyon, France

**Keywords:** Spine surgery, Exoscope, Meta-analysis, 3D, Cervical spine surgery, Lumbar spine surgery

## Abstract

Three-dimensional (3D) exoscopes have recently emerged as an alternative to the operating microscope (OM) in spine surgery, offering potential ergonomic and educational advantages. However, comparative evidence on surgical outcomes remains fragmented across different spinal pathologies and procedures,. With this work, we aim at comparing surgical and clinical outcomes of exoscopic versus microscopic spine surgery for degenerative conditions through a global meta-analysis, with procedure-specific subgroup analyses. A systematic review and meta-analysis were conducted according to PRISMA guidelines. Comparative studies evaluating 3D exoscope versus microscope in spine surgery for degenerative conditions were included. Continuous outcomes (operative time, blood loss, length of stay, visual analogue scale [VAS] scores) were pooled using a random-effects inverse-variance model and expressed as mean differences (MD). Dichotomous outcomes (complications) were analyzed using Mantel–Haenszel odds ratios (OR). Heterogeneity was quantified with I² and τ² statistics. Subgroup analyses were performed by procedure (ACDF, MIS-TLIF, lumbar microdecompression/microdiscectomy), along with sensitivity analyses and meta-regressions when indicated. A total of 785 patients across 7 studies were included. No significant differences were found in operative time (MD − 5.9 min; 95% CI − 16.4 to + 4.7), complications (OR 0.83; 95% CI 0.53–1.30), length of stay (MD − 0.15 days; 95% CI − 0.84 to + 0.53), or postoperative VAS scores. Blood loss was slightly reduced with the exoscope (MD − 5.9 mL; 95% CI − 11.1 to − 0.6), although the clinical relevance appears negligible. Subgroup analyses showed consistent findings across ACDF, MIS-TLIF, and lumbar microdecompression. Despite significant limitations, current literature supports comparable surgical and clinical outcomes between 3D exoscopes and OM across spine procedures. Exoscopes may offer ergonomic and didactic advantages without increasing operative time, complications, or hospital stay. However, standardized quantification of performance in the former non-clinical domains is missing. Further high-quality randomized studies are needed to confirm these findings and define procedure-specific benefits.

**Supplementary Information:**

The online version contains supplementary material available at 10.1007/s10143-026-04275-0.

## Introduction

Many of the most common spinal procedures, particularly with the growing adoption of minimally invasive surgery (MIS) techniques, are performed through narrow operative corridors that significantly limit visualization. To address this, operative microscopes (OM) are widely, though not universally, employed, to improve magnification and illumination. In recent years, three-dimensional (3D) exoscopes have emerged as a promising alternative, with reported advantages including improved ergonomics, greater maneuverability, and value in surgical teaching and team involvement [18, 35, 39]. Although the learning curve for exoscopes is often described as relatively short, there remains a paucity of evidence directly comparing their performance in spine surgery with that of traditional OM. Specifically, evidence on important intraoperative outcomes, such as operative time, blood loss, and other parameters potentially influenced by the choice of magnification device, remains limited. Furthermore, existing reviews are often incomplete or combine heterogeneous pathologies and techniques, introducing potential sources of bias. Furthermore, comparative evidence on individual spine techniques is limited to small series and lacks systematization.

The aim of this study is therefore to systematically synthesize the available evidence comparing 3D exoscopes and traditional OM in spine surgery for degenerative conditions. To reduce heterogeneity and better reflect real-world clinical practice, the analysis is limited to commonly performed procedures (ACDF, MIS-TLIF, lumbar microdiscectomy/microdecompression), with procedure-specific sub-analyses.

## Materials and methods

### Study design and review question

We registered the protocol of this study in PROSPERO (ID: CRD420251156505) on September 27, 2025. Study design followed the Preferred Reporting Items for Systematic reviews and Meta-Analyses (PRISMA) guidelines [31]. The review questions were formulated according to the population, intervention, comparison, and outcome (PICO) model:

#### Population

Adult patients (≥ 18 years) affected by degenerative spine conditions.

#### Intervention

Surgical treatment of the condition using a 3D exoscope.

#### Comparison(s)

Surgical treatment of the condition using a traditional OM.

#### Outcome(s)

Operative time, intraoperative blood loss (primary outcomes); length of hospital stay (LOS), surgical complications and VAS (Visual Assessment Scale) scores for postoperative axial and radicular pain (secondary outcomes). Descriptive evaluation of outcomes related to surgical comfort, image quality, didactic value of the instrument.

### Literature search

A literature search was performed in **PubMed/MEDLINE**,** Scopus and Web of Science**. Date of last search was September 30, 2025. The search strategy combined MeSH terms (for PubMed) and free-text words related to “spine surgery” and “exoscope” as follows: *((“Exoscopes“[All Fields] OR “Exoscope“[All Fields] OR “3D exoscope“[All Fields]) AND (“Spine“[All Fields] OR “Spinal Fusion“[MeSH Terms] OR “Laminectomy“[MeSH Terms] OR “Diskectomy“[MeSH Terms] OR “Spinal Diseases/surgery“[MeSH Terms] OR “Anterior Cervical Discectomy and Fusion“[All Fields] OR “ACDF“[All Fields]))*. The Scopus and Web of Science string was Spine AND Exoscop*. Reference lists of relevant reviews and included studies were also screened for forward search. Only English language articles were considered.

### Eligibility criteria

**Inclusion criteria** were:

- Studies comparing exoscope-assisted versus OM-assisted spine surgery.
- RCTs or observational studies (prospective or retrospective) reporting at least one outcome of interest.
- Studies including adult patients undergoing procedures to treat degenerative spine conditions.

**Exclusion criteria** were:

- Case reports, studies with < 10 patients, conference abstracts, reviews.
- Studies on cadavers/simulators/non-human animals.
- Studies without a comparator group.
- Studies not reporting extractable quantitative data.
- Studies not providing details on the specific spine procedures performed.
- Studies dealing exclusively or in part with complex pathologies (intra- or extra-dural tumors, intradural pathology, craniovertebral junction pathology, deformity, complex multilevel conditions) and/or complex procedures (corpectomies, deformity correction/osteotomies, antero-posterior surgeries).
- Studies dealing with mixed cranial and spinal pathologies.

### Risk of bias assessment

Risk of bias in observational studies was assessed using the **Newcastle–Ottawa Scale (NOS)**; for RCTs, the **Cochrane Risk of Bias 2 tool (RoB 2)** was used instead. Discrepancies between reviewers were resolved by consensus with the senior author. The risk of publication bias was considered with funnel plots; when funnel plots could not be generated, publication bias was assessed narratively.

### Data extraction and collected variables

For each included study, two independent reviewers extracted the following information: study design, sample size, type of procedure, baseline demographics, and outcome measures.

Collected outcomes were divided into:

- **Continuous variables**: operative time, intraoperative blood loss, hospital stay, visual analog scale (VAS) for axial and radicular pain (pre- and postoperative), and age (for baseline comparability).
- **Binary outcomes**: intra- or postoperative complications (any among unintended durotomy, hematoma, infection, nerve damage, malpositioning of material).

When studies reported results for multiple surgical procedures (e.g., ACDF and TLIF, or ACDF and lumbar decompression), these were considered separately in the analyses ad independent study-groups, as the sub-populations of patients were distinct.

### Statistical analysis

Meta-analyses were performed using the **R** “**meta” package**. For continuous outcomes, mean differences (MD) with 95% confidence intervals (CI) were calculated using the **inverse variance method**. For dichotomous outcomes, odds ratios (OR) with 95% CI were computed using the **Mantel–Haenszel method**. Random-effects models were applied using the **restricted maximum-likelihood estimator (REML)**, with Hartung–Knapp adjustment for confidence intervals. Heterogeneity was quantified with the **I² statistic** and Cochran’s Q test. Sensitivity analyses were conducted using leave-one-out methods to assess the influence of individual studies on the overall findings.

Subgroup analyses were performed according to the **surgical procedure (ACDF**,** MIS-TLIF**,** lumbar microdecompression/microdiscectomy)** by including procedure type as a stratification variable. When a subgroup was represented by only one study, results were presented descriptively but interpreted cautiously.

All analyses were performed separately for global effects and procedure-specific subgroups. Statistical significance was defined as *p* < 0.05.

When standard deviations were not reported for mean values, the parameter (or the study, if they were not available for any outcomes) were excluded from analysis.

## Results

### Results of the literature search

**Fig. 1:**
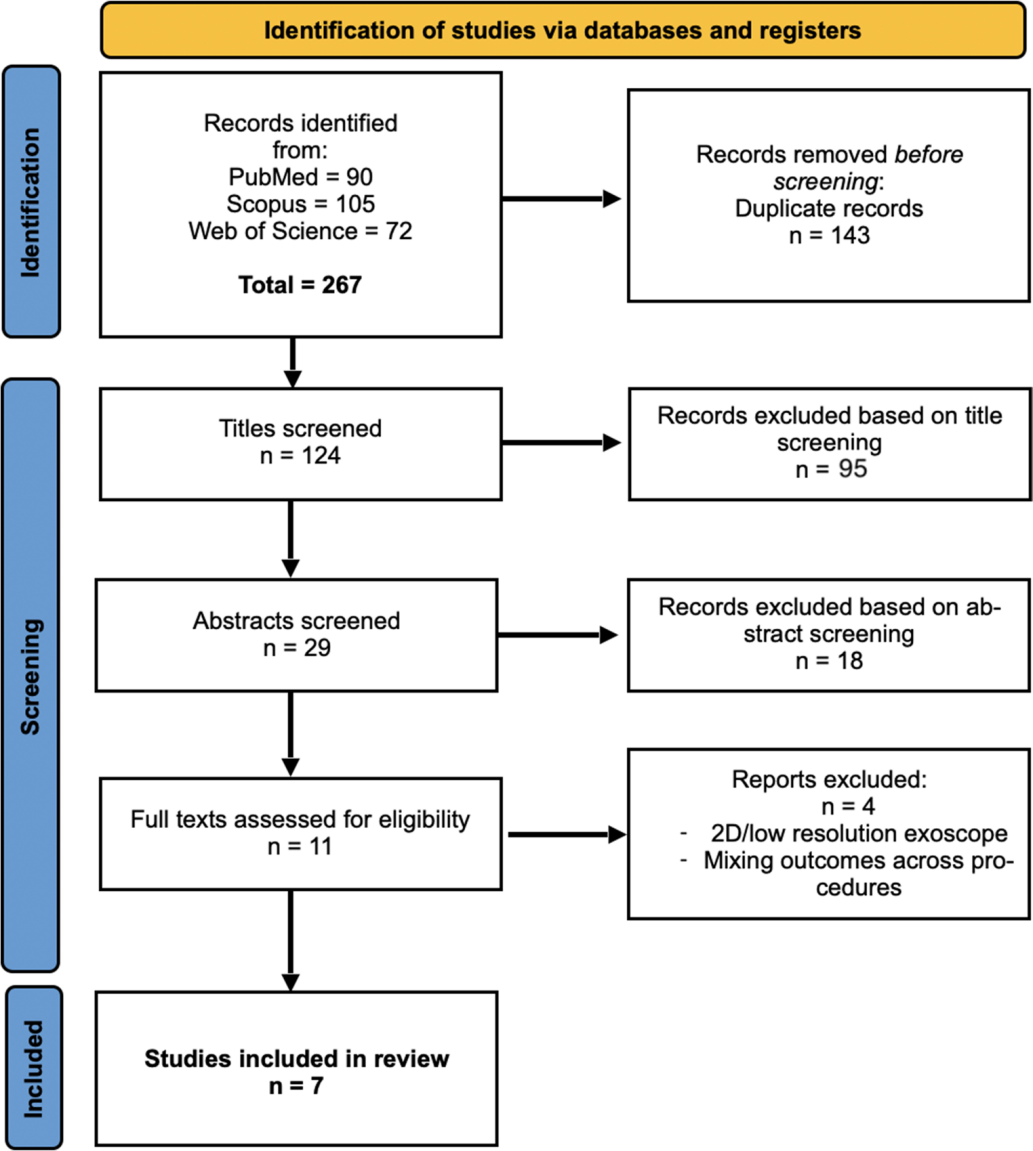
PRISMA diagram of the literature search

The systematic search yielded a total of 267 studies. After removal of duplicates (*n* = 143) and title screening (*n* = 124), the abstracts of 29 papers were assessed, of which 11 were chosen for full-text review and review of bibliographies (Fig. 1). Finally, 7 papers were included in the meta-analysis [17, 23, 24, 36, 38, 41, 42]. These studies were published between 2020 and 2024. **For the purpose of our analysis**,** we considered the procedure-subgroups in each study as individual studies**,** bringing the number of included studies to 10**. These are indicated as “studies-groups”. This was statistically appropriate as (1) subgroup populations were independent in all cases; (2) studies often did not report global but only subgroup-associated means and standard deviations, preventing their inclusion in the analysis as a whole.

### Risk of bias assessment

We applied the Newcastle-Ottawa scale to rate the included papers. All studies were rated as good quality (> 6 stars). Ratings of the individual studies are reported in Table 1.

**Table 1:** Newcastle-Ottawa scale ratings of the included studies. Numbers represent how many stars were assigned to each study in the various domains

Selection	Siller 2020 [38]	Lin 2021 [24]	Yao 2021 [41]	Yao 2022 [42]	Lin 2023 [23]	Sarikonda 2023 [36]	Innocenti 2024 [17]
	4	4	4	4	4	4	4
Comparability	2	2	2	2	2	1	1
Outcomes	3	3	3	3	3	3	3
Total	9	9	9	9	9	8	8

### Demographics and study features

Data reported in the included studies were as follows:

#### Patients

The total number of patients across studies was 785 (333 in the exoscope group and 452 in the OM group). Baseline demographics (age and sex distribution) were comparable between the two groups (Table 2). Mean age ranged between 51.6 and 61.9 years across cohorts.

**Table 2:** Summary of the demographics, follow-up length and non-clinical features of the included studies. FU, follow-up; NR, not reported. “Decompression” stands for lumbar microdecompression or microdiscectomy

Author, year	Group	*N*	Mean age	Female *N*	Mean levels	FU (months)	Surgeons (*N*)	Exoscope
Siller, 2020, ACDF [38]	M	20	56.7 ± 17.9	7	1.0	NR	3	Vitom 3D
	E	20	57.5 ± 10.8	7	1.0	NR	3	
Lin, 2022, ACDF [24]	M	27	51.6 ± 10.5	12	1.0	20.7	2	Mitaka Kestrel View II
	E	23	52.2 ± 10.6	11	1.0	19.3	2	
Yao, 2021, ACDF [41]	M	27	53.0 ± 10.3	10	1.6	16.3	1	Mitaka Kestrel View II
	E	21	54.0 ± 8.5	8	1.6	16.4	1	
Innocenti, 2024, ACDF [17]	M	59	NR	NR	1.5	NR	Multiple	Orbeye II
	E	52	NR	NR	1.5	NR	Multiple	
Sarikonda, 2024, ACDF [36]	M	52	60.3 ± 12.9	24	1.4	NR	NR	NR (3D exoscope)
	E	26	56.6 ± 9.1	14	1.8	NR	NR	
Yao, 2022, TLIF [42]	M	25	56.9 ± 9.7	11	1.0	> 12	1	Mitaka Kestrel View II
	E	22	55.8 ± 7.9	9	1.0	> 12	1	
Lin, 2023, TLIF [23]	M	43	57.9 ± 8.6	18	1.0	18.8	Multiple	Mitaka Kestrel View II
	E	47	58.4 ± 10.5	20	1.0	19.2	Multiple	
Innocenti, 2024, TLIF [17]	M	46	NR	NR	1.5	NR	Multiple	Orbeye II
	E	33	NR	NR	1.5	NR	Multiple	
Siller, 2020, Decompression [38]	M	40	59.1 ± 15.2	24	1	NR	3	Vitom3D
	E	40	61.9 ± 15.0	26	1	NR	3	
Innocenti, 2024, Decompression [17]	M	113	NR	NR	1	NR	Multiple	Orbeye II
	E	49	NR	NR	1	NR	Multiple	
Total	M	452						
	E	333						

#### Pathologies and procedures

Conditions treated included: cervical radiculopathy and/or myelopathy, lumbar stenosis and/or radiculopathy due spondylosis or disc herniation. Procedures included single- or bi-level anterior cervical discectomy and fusion (ACDF), single- or bi-level level minimally invasive transforaminal lumbar interbody fusion (MIS-TLIF), and single-level lumbar microdecompression/microdiscectomy.

### Primary outcomes

A summary of the outcomes reported in each study is provided in Table 3.

**Table 3:** Summary of primary and secondary outcomes as reported by the included studies. LOS, length of hospital stay; NR, not reported; VAS, visual assessment scale

Author, year	Group	Operative time	Blood loss	LOS	Postop VAS axial	Preop VAS axial	Postop VAS radicular	Preop VAS radicular	Complications
Siller, 2020, ACDF [38]	M	116.0 ± 37.0	93.0 ± 67.0	7.3 ± 5.8	0.0 ± 0.0	5.0 ± 1.9	0.0 ± 0.7	5.0 ± 1.9	NR (no neurological complications
	E	132.0 ± 25.0	97.0 ± 83.0	5.9 ± 2.6	0.0 ± 0.0	5.0 ± 1.9	0.0 ± 0.7	5.0 ± 1.9	NR (no neurological complications
Lin, 2022, ACDF [24]	M	96.0 ± 12.0	64.0 ± 21.0	8.9 ± 4.1	1.3 ± 0.8	6.2 ± 1.4	NR	NR	None
	E	96.0 ± 11.0	58.0 ± 17.0	9.7 ± 4.8	1.3 ± 0.9	6.3 ± 1.2	NR	NR	None
Yao, 2021, ACDF [41]	M	129.9 ± 32.7	63.9 ± 20.7	5.6 ± 1.9	4.3 ± 0.8	5.6 ± 0.9	NR	NR	0
	E	111.2 ± 26.9	57.1 ± 20.7	5.6 ± 1.6	3.9 ± 0.8	5.9 ± 0.8	NR	NR	1
Innocenti, 2024, ACDF [17]	M	56.9 ± 19.6	NR	NR	NR	NR	NR	NR	C5 palsy (1)
	E	63.3 ± 14.9	NR	NR	NR	NR	NR	NR	Infection (1)
Sarikonda, 2024, ACDF [36]	M	146.0 ± 47.0	NR	NR	NR	NR	NR	NR	NR
	E	110.0 ± 34.0	NR	NR	NR	NR	NR	NR	NR
Yao, 2022, TLIF [42]	M	121.9 ± 16.9	63.3 ± 20.3	11.7 ± 2.5	3.0 ± 0.8	6.5 ± 1.1	2.2 ± 0.9	5.4 ± 1.6	Numbness (2), Screw loosening (1)
	E	111.0 ± 19.9	57.2 ± 20.2	11.1 ± 1.8	2.5 ± 0.7	6.7 ± 1.2	2.1 ± 0.6	5.1 ± 1.3	Screw loosening (2)
Lin, 2023, TLIF [23]	M	142.0 ± 36.1	122.1 ± 51.2	8.9 ± 4.1	4.2 ± 1.4	5.3 ± 1.5	4.1 ± 1.4	6.5 ± 1.5	NR
	E	146.7 ± 30.5	114.1 ± 52.9	9.7 ± 4.8	4.4 ± 1.5	5.2 ± 1.4	3.9 ± 1.5	6.4 ± 1.7	NR
Innocenti, 2024, TLIF [17]	M	140.8 ± 32.3	NR	NR	NR	NR	NR	NR	Durotomy (4)
	E	116.8 ± 33.2	NR	NR	NR	NR	NR	NR	Durotomy (3)
Siller, 2020, Decompression [38]	M	113.0 ± 37.0	109.0 ± 97.0	7.4 ± 7.3	2.0 ± 1.7	6.0 ± 2.0	1.0 ± 1.2	6.0 ± 2.0	NR (no neurological complications
	E	112.0 ± 33.0	155.0 ± 218.0	6.5 ± 1.8	0.0 ± 2.2	7.0 ± 2.2	0.0 ± 1.7	7.0 ± 2.2	NR (no neurological complications
Innocenti, 2024, Decompression [17]	M	51.5 ± 23.9	NR	NR	NR	NR	NR	NR	Durotomy (4), Hematoma (1)
	E	49.6 ± 13.0	NR	NR	NR	NR	NR	NR	Durotomy (2)

#### Operative time

**Fig. 2:**
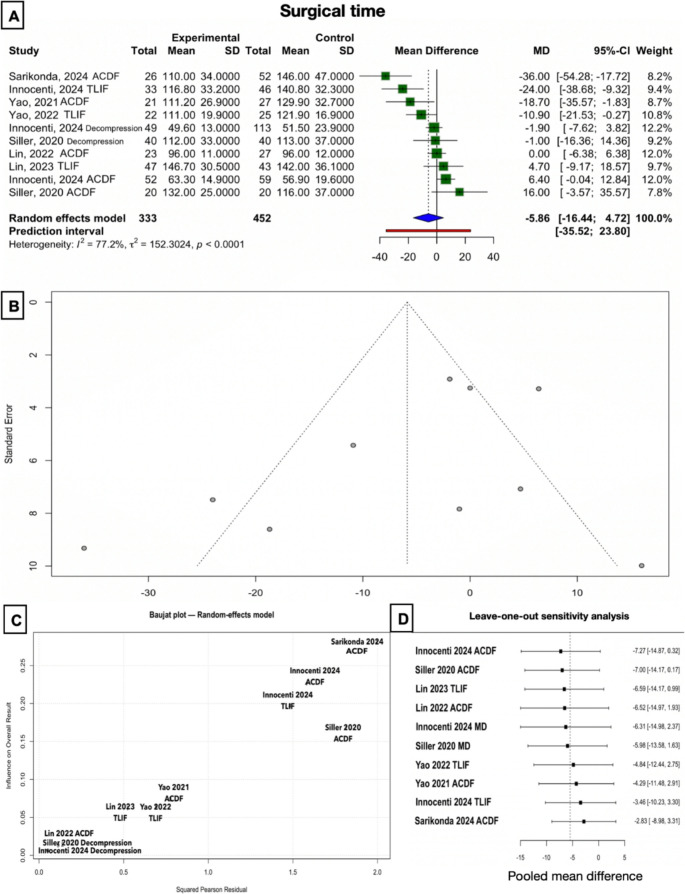
Plots for the surgical time outcome. (**A**) Forest plot of the global analysis, “experimental” is exoscope, while “control” is OM. (**B**) Funnel plot for the global analysis. (**C**) Baujat plot, identifying the study by Sarikonda 2024 on ACDF as the main outlier (top right corner) [36]. (**D**) Results of the leave-one-out sensitivity analysis, demonstrating that, however, overall conclusions are stable. Each row represents the calculated mean difference and confidence interval after leaving out the respective study. MD: lumbar microdecompression

Across 10 studies-groups, there was no statistically significant difference in surgery duration between exoscope and OM. The random-effects model showed a mean difference (MD) of −5.86 min (95% CI: −16.44 to 4.72, *p* = 0.242), indicating a non-significant trend toward shorter durations with the exoscope (Fig. 2). Heterogeneity was high (I² = 77.2%), suggesting substantial variability between studies-groups. None of the treatment subgroups showed significant differences, as well (Supplementary Fig. 1):

- **TLIF (3 studies)**: MD = −9.96 [−44.90, + 24.97], moderate heterogeneity (I² = 74.5%).
- **Decompression (2 studies)**: MD = −1.79 [−5.53, + 1.95], no heterogeneity.

Sensitivity analyses were performed to assess the robustness of the findings (Fig. 2** C **and** D**). Leave-one-out analysis showed that no single study substantially altered the overall effect, with pooled estimates consistently non-significant. Influence diagnostics identified Sarikonda et al. (2024) as the most influential study, contributing disproportionately to heterogeneity (I² reduction from 72.6% to 67.1% upon exclusion) [36]. However, the exclusion of this study did not materially change the effect estimate (Hedges’ g = 0.10, 95% CI − 0.17 to 0.38, *p* = 0.45), indicating that the overall conclusions are stable.

Meta-regression analysis was performed to assess whether the type of procedure (ACDF, TLIF, decompression) influenced operative time differences between exoscope and OM (Supplementary Fig. 1). No significant effect of procedure type was observed (QM (2) = 0.66, *p* = 0.72), and the moderator explained none of the residual heterogeneity (R² = 0%). Residual heterogeneity remained high (I² = 76%), indicating substantial variability across studies that was not accounted for by procedural subgroup. These findings suggest that the (lack of) differences in operative time between exoscope and OM are consistent across procedural types.

#### Intraoperative blood loss

**Fig. 3:**
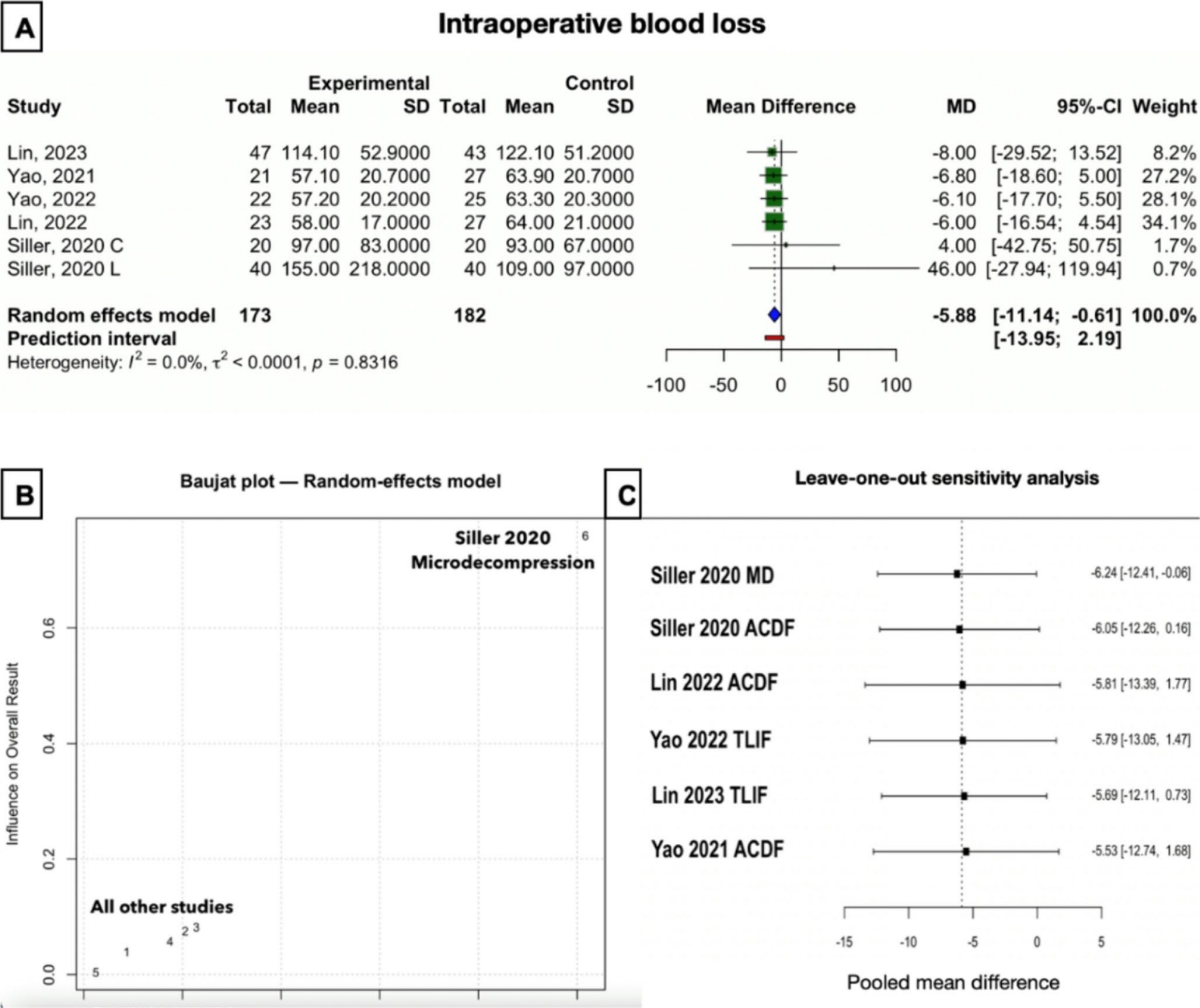
Plots for the intraoperative blood loss outcome. (**A**) Forest plot of the global analysis, “experimental” is exoscope, while “control” is OM. (**B**) Baujat plot. (**C**) Plot of the leave-one-out sensitivity analysis. Each row represents the calculated mean difference and confidence interval after leaving out the respective study. MD: lumbar microdecompression

Across 6 studies-groups, there was a statistically significant reduction in blood loss with the exoscope (MD − 5.9 mL; 95% CI − 11.1 to − 0.6; *p* = 0.035) (Fig. 3). However, the entity of the difference is clinically irrelevant. Heterogeneity was negligible (I² = 0%). This was confirmed by subgroup analysis (Supplementary Fig. 2):

- **ACDF (3 studies)**: MD = −6.07 [−11.36, −0.78], no heterogeneity.
- **TLIF (2 studies)**: MD = −6.53 [−0.16.61, + 3.56], no heterogeneity.
- **Decompression (1 study)**: MD = + 46.00 [-27.94; 119.94], single-study observation, no generalization possible. Of note, given the large standard deviation, this study was attributed a low weight in the overall analysis.

In spite of the lumbar microdecompression subgroup from the study by Siller et al. (2020) being localized in the top right corner of Baujat plot due to its significantly contrasting findings, the leave-one-out sensitivity analysis for blood loss showed that no single study had a disproportionate influence on the overall result [38] (Fig. 3). This strengthens confidence that the conclusion (significant but clinically irrelevant difference in blood loss between exoscope and OM) is robust.

### Secondary outcomes

#### Pain

**Fig. 4:**
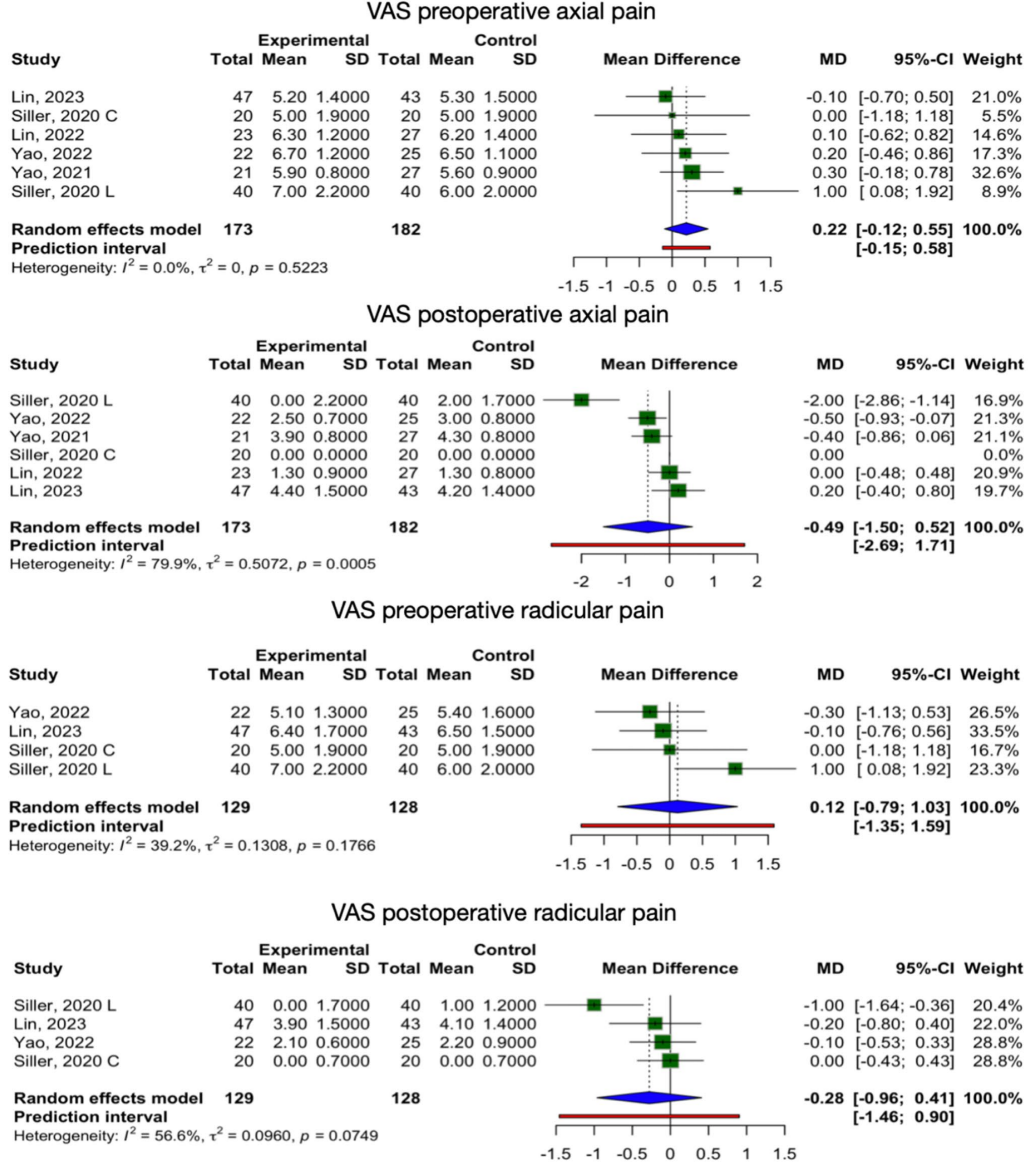
Forest plot of the global assessment of pain outcomes. “Experimental” is exoscope, while “control” is OM

VAS for postoperative pain, both axial (6 studies-groups) and radicular (4 studies-groups), did not reveal significant global differences between exoscope and OM groups (axial pain mean difference = −0.49, 95% CI: −1.50 to 0.52, I² = 79.9%; radicular pain mean difference: −0.28, 95% CI: −0.96 to 0.41, I² = 56.6%) (Fig. 4).

Importantly, pre-operative pain was comparable between groups (axial pain mean difference = 0.22, 95% CI: −0.12 to 0.55; radicular pain mean difference = 0.12, 95% CI: −0.79 to 1.03). Subgroup analyses also showed overlapping confidence intervals across procedures (Supplementary Fig. 3). Notably, for pain outcomes, only one study was available in the decompression subgroup, which limits interpretation and prevents meaningful subgroup comparisons.

#### Postoperative complications

**Fig. 5:**
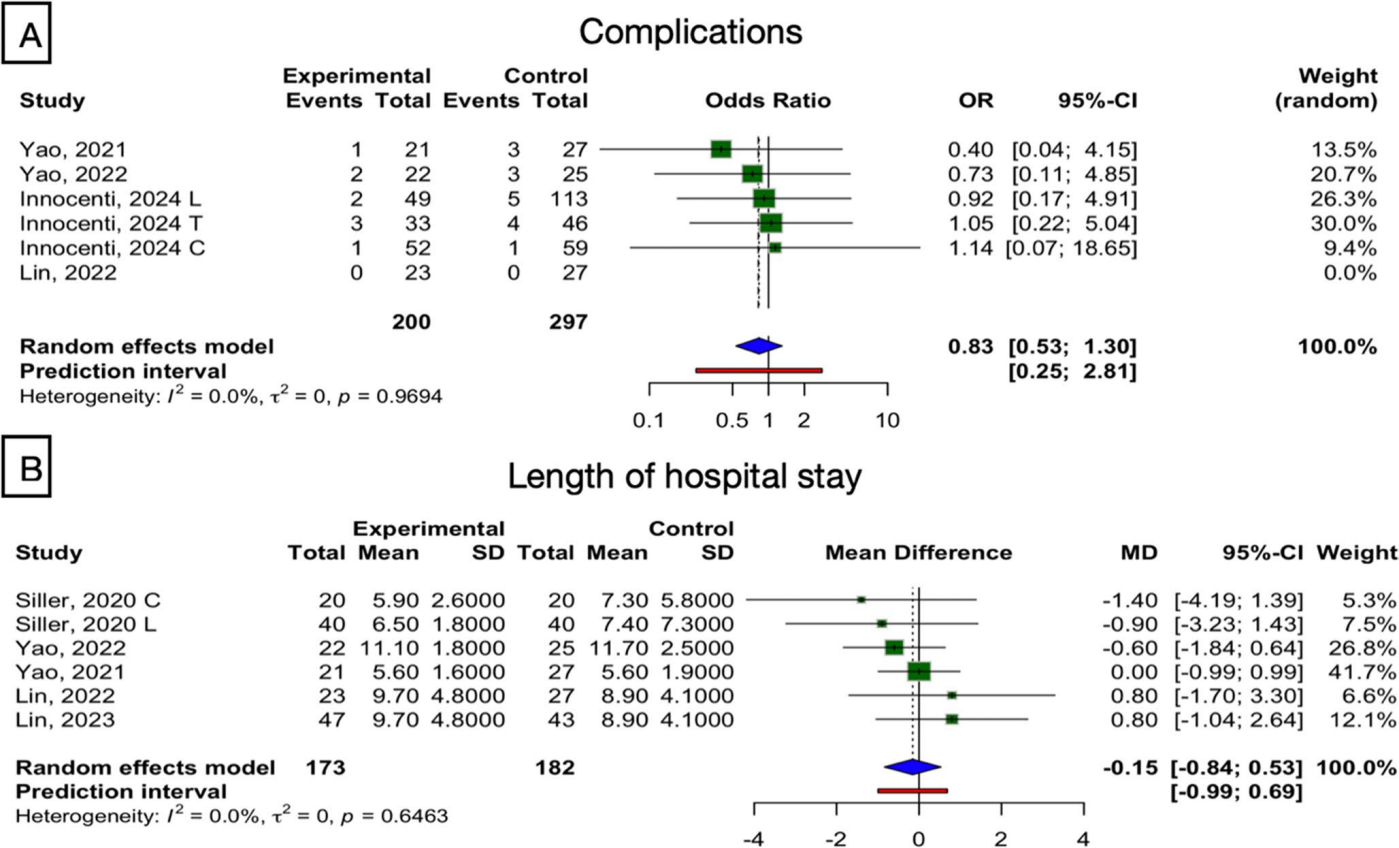
(**A**) Forest plot of the global assessment of complications. (**B**) Forest plot of the global assessment of length of hospital stay.

Five studies-groups reported on complications. The pooled analysis found no significant difference between exoscope and OM in terms of complication rates (random-effects OR 0.83; 95% CI 0.53–1.30; *p* = 0.32; I² = 0%) (Fig. 5A).

In subgroup analyses, ACDF (OR 0.62) and TLIF (OR 0.91) each showed no significant differences between techniques, and between-group comparisons confirmed the absence of subgroup effects (*p* for interaction > 0.7). The decompression subgroup was represented by a single study is thus not considered in subgroup analysis.

#### Length of hospital stay

Among 6 studies-groups, the overall mean difference was very small and non-significant (MD = −0.15 days, 95% CI: −0.84 to 0.53, *p* = 0.591), with no heterogeneity (I² = 0%), indicating consistent findings across studies (**Fig. 5B**). Mean differences in treatment subgroups were small and non-significant: ACDF (3 studies): MD = −0.04 [−1.62, 1.54]; TLIF (2 studies): MD = −0.07 [−1.19, 0.86], Decompression (1 study): single-study observation.

### Qualitative description of non-clinical parameters

Parameters such as surgeon’s comfort, maneuverability, learning curve and didactical value were assessed with scales and questionnaires that differed across studies, so a quantitative analysis is not possible. We thus performed a qualitative assessment by selecting homogeneous domains that were reported in the studies with minimal differences: comfort of the operators, maneuverability, general handling, efficiency of focus/optical adjustment, image/video quality, depth perception, illumination, didactic value, and experience of wearing 3D glasses; for each domain, we determined the number of studies where the performance of the exoscope was considered to be superior, equal or inferior to the OM, respectively (Supplementary Table 1). When a study used multiple indicators to represent different aspects of one domain, a mean of the score across the indicators was used to represent overall performance in that domain. Overall, sufficient data were available in four of the included studies (Siller et al., 2020, Lin et al., 2022; Yao et al., 2022; Lin et al., 2023 [23, 24, 38, 42]. The exoscope was consistently described as superior to OM as concerns operator comfort and value for didactics and team involvement. Optical parameters, video quality, illumination and handling received a more mixed rating. The necessity to wear 3D glasses was consistently reported as a drawback as compared to OM.

## Discussion

Neurosurgery and spine surgery are often defined by narrow operative corridors and the need to work on small structures near to delicate neural elements. To improve safety, precision, and surgical outcomes, magnification devices are commonly employed. Traditionally, operating microscopes have been the standard in this field, offering some advantages over loupes, including adjustable magnification, superior illumination, and higher image resolution. However, the bulky design of OM requires positioning directly over the operative field and looking through oculars, which can restrict the surgeon’s freedom of movement and force a constant compromise between adjusting the OM and adapting the operator’s position [1].

In the past decade, exoscopes have affirmed across all domains of neurosurgery and spine surgery as an alternative technology that keeps the main benefits of OM (magnification, clarity, and illumination) while potentially addressing many of their limitations [4, 6, 7, 11, 26, 27, 34, 40]. Even if a (short) learning curve is necessary when moving to exoscopes from OM, since with the former the surgeon is not looking at the field through binoculars but via a screen, the device presents many potential advantages. Indeed, exoscopes are less bulky, occupy reduced space above the surgical field, and are reported to offer easier maneuverability [5, 13, 14, 21, 27]. Moreover, they could improve operator comfort by allowing for greater mobility and less constrained head and neck posture [20]. Additional reported benefits include improved depth perception and enhanced educational value, as the 3D visualization can be shared with the entire surgical team, promoting better teaching and team participation [9, 34].

In addition to the suggested ergonomic, didactic and technical advantages, 3D exoscopes may influence relevant surgical and clinical parameters such as surgical time and intraoperative blood loss (with some authors claiming that better visualization of the entire surgical field would allow for more effective recognition and control of active bleeding sites) [35]. Other outcomes may likewise be affected, such as improvement of radicular pain (due to better, or worse, visualization and decompression of nerve roots), axial pain (due to less manipulation of soft tissues and muscle), and rates of complications (for example, infections, by avoiding non-sterile contact with the operator’s face as happens with OM). Our meta-analysis (785 patients, 7 studies) showed overall equivalence between the exoscope and the operating microscope for these clinical outcomes. Operative time did not differ significantly (MD − 5.9 min; 95% CI − 16.4 to + 4.7), nor did length of hospital stay (MD − 0.15 days; 95% CI − 0.84 to + 0.53), postoperative axial pain (MD − 0.49; 95% CI − 1.50 to + 0.52), radicular pain (MD − 0.28; 95% CI − 0.96 to + 0.41), or complication rates (OR 0.83; 95% CI 0.53–1.30). A statistically significant but clinically negligible reduction in blood loss was observed with the exoscope (MD − 5.9 mL; 95% CI − 11.1 to − 0.6).

Although exoscopes have been available for more than a decade high-quality comparative evidence of their performance as compared to traditional OM in spine surgery remains limited [25, 37]. Most series in the literature are non-comparative [3, 28]. While comparative studies exist, many include heterogeneous pathologies and/or procedures, which introduces significant bias [8, 11, 30]. Nevertheless, a few comparative series focusing on single procedures (or reporting subgroup data for individual procedures) have been published [17, 23, 24, 36, 38, 41, 42].

Systematic reviews and meta-analyses have previously addressed this topic. However, these are either (1) narrative/qualitative assessments [13, 18, 27, 34, 35, 39] or (2) incorporated studies with mixed spinal pathologies or surgical techniques, included 2D exoscopes, or compared exoscopes to heterogeneous magnification tools, such as surgical loupes, thereby introducing significant variability and potential biases [2, 5, 12, 22, 29, 32]. By contrast, the aim of our analysis was to (1) include only comparative studies of commonly performed spine surgeries, excluding pathologies (e.g., tumors, craniovertebral junction conditions, deformity) and techniques (e.g., corpectomies, osteotomies, etc.) that could confound outcomes such as intraoperative blood loss, surgical time, and clinical results; and (2) include only single-technique studies, or those reporting technique-specific outcomes [10, 11, 15, 16, 19, 33]. Consequently, our dataset focused on procedures with minimal blood loss and fairly uniform duration in experienced hands (ACDF, MIS-TLIF, lumbar microdecompression). To further reduce the impact of inter-technique heterogeneity, we placed strong emphasis on subgroup and sensitivity analyses to evaluate potential biases linked to specific techniques or individual studies. We also noted the number of levels treated on average in each study subgroup (single level in 4, and 1–2 levels in 3), to make sure this was not a relevant source of heterogeneity (Table 1).

For the purpose of our analysis, we have subdivided studies reporting on multiple techniques as if each technique was a separate study. This is methodologically viable as the patient populations were different. Our analysis demonstrated that operative time did not differ significantly between exoscope and OM use across ten studies-groups, with a non-significant trend toward shorter procedures in the exoscope group. Subgroup analyses for ACDF, TLIF, and decompression procedures were consistent with the overall findings, though heterogeneity was high (particularly for ACDF and TLIF) suggesting variability across study designs and populations. Sensitivity analyses confirmed that no single study, aside from Sarikonda et al., substantially influenced results, reinforcing the presence of widespread rather than study-specific heterogeneity [36]. In contrast, intraoperative blood loss was consistently lower in the exoscope group, reaching statistical significance; however, the absolute difference was minimal (≈ 6 mL) and thus clinically negligible. Notably, this outcome showed negligible heterogeneity, despite Siller et al. (2020) reporting significantly higher blood loss in the exoscope group for lumbar microdecompression [38]. This is likely due to the low weight of that study in the overall analysis, caused by its large confidence interval and standard deviation. Regarding secondary outcomes, no significant differences were observed between groups for postoperative pain, complication rates, or length of hospital stay. Pain analyses showed substantial heterogeneity, but subgroup and sensitivity assessments revealed no meaningful divergence between techniques. We made a distinction between radicular and axial pain, as postoperative radicular pain could be interpreted as an indirect measure of the efficacy of nerve root decompression, which could be impacted by better visualization. Complication rates and hospital stay were remarkably consistent across studies, with no heterogeneity and overlapping confidence intervals across subgroups. Taken together, these findings suggest that, while exoscopes may offer small advantages in blood loss and operative time under specific conditions, the overall clinical equivalence with OM remains clear across commonly performed spinal procedures.

**Fig. 6:**
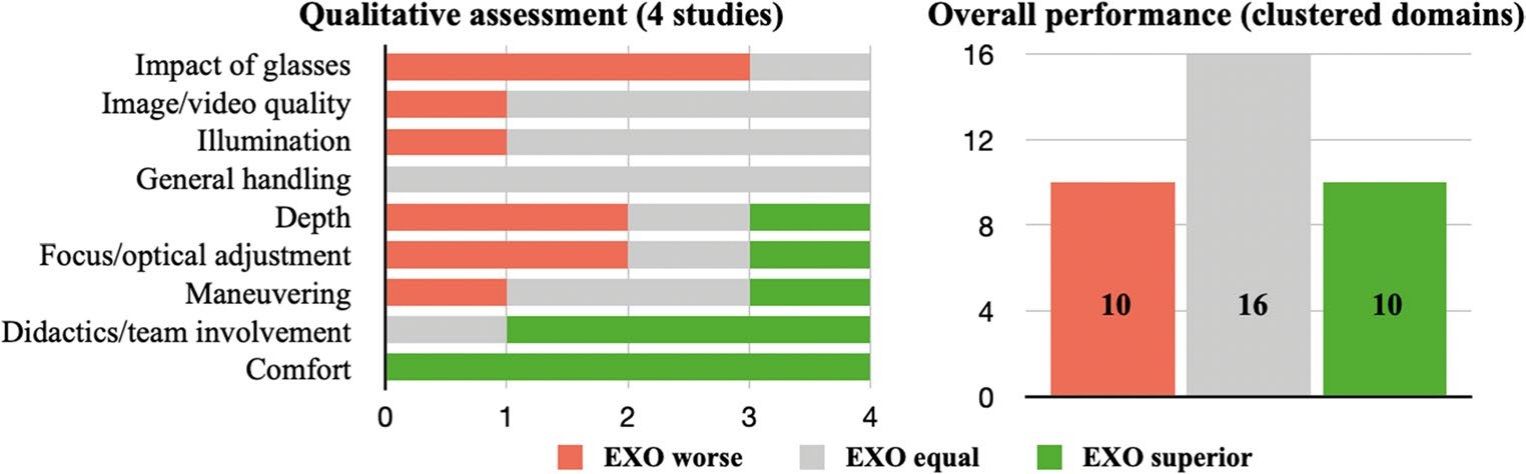
Graphical representation of the qualitative assessment of the performance of Exoscope vs. OM across different nonclinical domains. The “Overall performance” plot stacks the individual cells across all domains, based on performance. EXO: 3D exoscope

Most reported advantages of 3D exoscopes are connected to non-clinical factors, such as surgeon comfort, ease of maneuverability and handling, and their value in engaging the operating room team and surgical trainees [5, 9, 14, 27, 34]. However, there is currently no standardized method for evaluating these parameters, which makes formal quantitative meta-analysis unfeasible. Despite this limitation, four of the included studies assessed these domains in a reasonably consistent manner. We therefore synthesized recurring themes (surgeon comfort, image quality, didactic value, the impact of 3D glasses, and others) and classified exoscope performance relative to OM as worse, equal, or superior (Fig. 6). Overall, the exoscope was consistently favored over the OM for surgical comfort as well as its didactic and team-involvement benefits. Handling and maneuverability were generally rated as comparable to those of the OM. Optical qualities, including illumination, focus, image quality, and depth perception, were reported as comparable or slightly inferior to the OM. The least appreciated aspect across studies was the mandatory use of 3D glasses, which was consistently cited as a drawback. When clustering all individual cells-domains across studies, the exoscope was rated superior on 10 occasions, equivalent on 16 occasions, and inferior on 10 occasions (Fig. 6). These results align with broader narrative assessments published previously, which consistently highlighted superior ergonomics and didactic value, while reporting more mixed findings regarding image quality, depth perception, and handling [5, 27].

This meta-analysis presents several limitations. First, the number of available studies was relatively small, particularly for some surgical subgroups such as lumbar microdecompression, where only one trial contributed to several outcomes. This restricts the ability to draw robust subgroup comparisons when it comes to this technique and limits the statistical power of the analyses. However, our total sample size for any outcomes was superior or comparable to that of other reviews that had less selective inclusion criteria [5, 18, 39]. Second, although most outcomes showed low or no heterogeneity, the outcome of surgical duration was marked by substantial heterogeneity. Sensitivity analyses suggested that the overall findings for duration were influenced disproportionately by a single study (Innocenti et al., 2024), highlighting the vulnerability of the results to outlier effects [17]. However, the leave-one-out strategy demonstrated that results would not change significantly when those studies were excluded. Third, variability in study designs, sample sizes, and surgical populations may have introduced clinical heterogeneity that could not be fully accounted for, despite subgroup analyses by surgical procedure. In particular, although the analysis was deliberately restricted to common degenerative procedures typically involving one or two levels in order to reduce clinical variability, residual heterogeneity cannot be completely excluded, as potentially relevant variables (such as exact number of treated levels, primary versus revision status, and case-specific complexity) were not consistently reported across studies and therefore could not be formally incorporated into the pooled analyses. Fourth, outcome definitions and reporting were not always standardized across studies, particularly for complications, which may have introduced reporting bias. Fifth, while risk of bias was assessed, the limited number of studies in each subgroup makes it difficult to formally explore publication bias or small-study effects. Moreover, as with any surgical series describing new techniques or technologies, a risk of bias related to preferential publication of positive results can not be excluded. Finally, we did not include a cost analysis in our paper, due to insufficient availability of data. Cost analyses would be fundamental to determine whether the potential advantages offered by exoscopes are indeed cost-effective.

These limitations indicate that the conclusions of this meta-analysis should be interpreted with caution and underscore the need for larger, prospective comparative studies with, ideally, more standardized reporting of both clinical and non-clinical (comfort, didactic value, etc.) parameters.

In conclusion, our meta-analysis indicates that the use of 3D exoscopes in common spine surgeries for degenerative conditions (ACDF, MIS-TLIF, and lumbar microdecompression/microdiscectomy) provides outcomes comparable to traditional operative microscopes in terms of surgical time, complication rates, length of hospital stay, and postoperative pain. A statistically significant reduction in intraoperative blood loss was observed with exoscopes, but the absolute difference was clinically negligible. Qualitative assessments consistently highlighted advantages in ergonomics and educational value, while feedback on optical quality, maneuverability, and depth perception was mixed; the mandatory use of 3D glasses emerged as the main drawback. These findings should be interpreted with caution given the limited sample sizes, heterogeneity in study designs, and variability in outcome reporting. Larger, prospective trials are warranted to validate these results, better define procedure-specific benefits, and provide quantitative assessments of technical and ergonomic parameters.

## Supplementary Information

Below is the link to the electronic supplementary material.

Supplementary Material 1 (PDF 3.42 MB)

## Data Availability

No datasets were generated or analysed during the current study.
