# Supplementary material for: Exoscope versus microscope in spine surgery: A meta-analysis based on procedure-specific subgroup analysis
Source: Neurosurg Rev. 2026 Apr 10;49(1):345. doi: 10.1007/s10143-026-04275-0 (PMC13068744; doi:10.1007/s10143-026-04275-0)
Supplement: Supplementary file 1 — Supplementary Material 1 (PDF 3.42 MB) [file 10143_2026_4275_MOESM1_ESM.pdf]

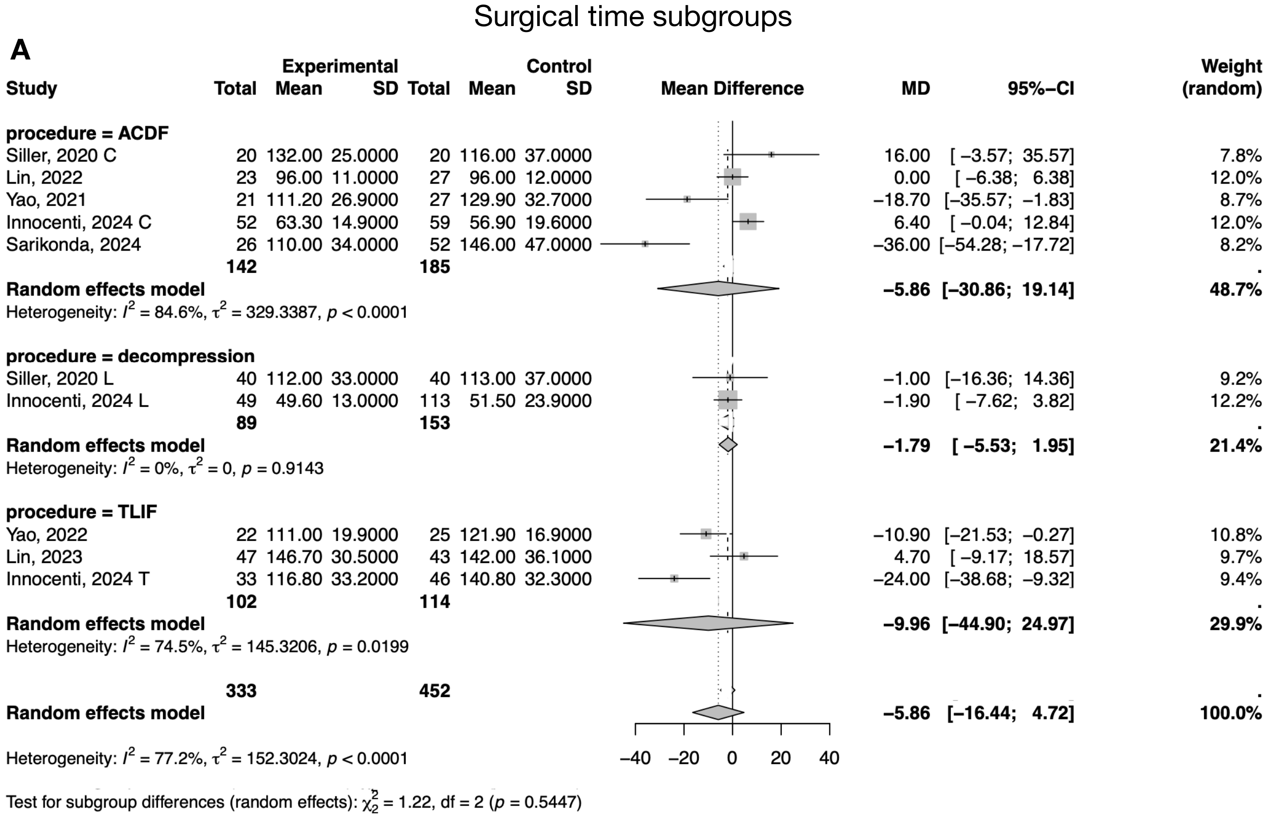

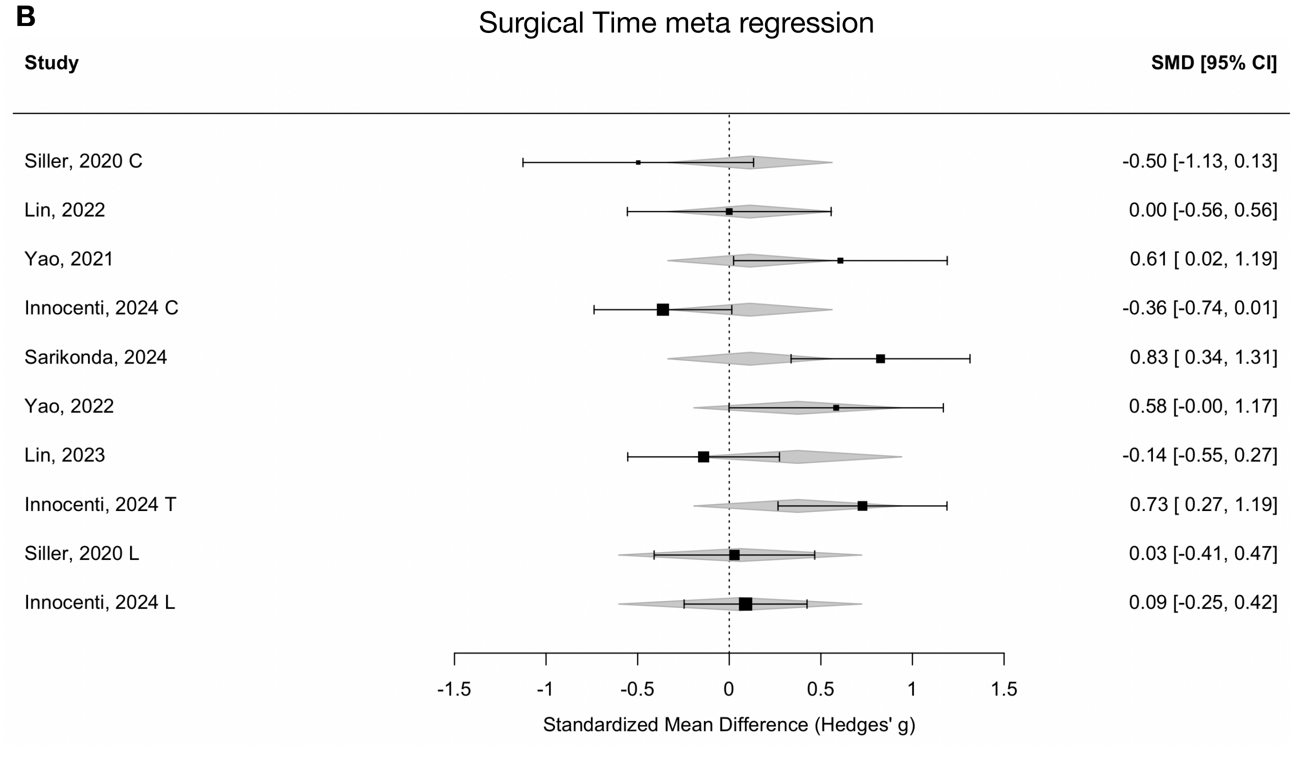

Supplementary figure 1. A) forest plot of the subgroup analysis for the outcome “surgical time”. B) Results of the meta regression analysis for the same outcome

Intraoperative blood loss subgroups

Study	Experimental Total	Experimental Mean	Experimental SD	Control Total	Control Mean	Control SD	MD	95%-CI	Weight (random)
**procedure = ACDF**									
Siller, 2020 C	20	97.00	83.0000	20	93.00	67.0000	4.00	[−42.75; 50.75]	1.7%
Lin, 2022	23	58.00	17.0000	27	64.00	21.0000	−6.00	[−16.54; 4.54]	34.1%
Yao, 2021	21	57.10	20.7000	27	63.90	20.7000	−6.80	[−18.60; 5.00]	27.2%
**Random effects model**							**−6.07**	**[−11.36; −0.78]**	**63.0%**
Heterogeneity: $I^2 = 0\%$, $\tau^2 = 0$, $p = 0.9080$									
**procedure = decompression**									
Siller, 2020 L	40	155.00	218.0000	40	109.00	97.0000	46.00	[−27.94; 119.94]	0.7%
**procedure = TLIF**									
Yao, 2022	22	57.20	20.2000	25	63.30	20.3000	−6.10	[−17.70; 5.50]	28.1%
Lin, 2023	47	114.10	52.9000	43	122.10	51.2000	−8.00	[−29.52; 13.52]	8.2%
**Random effects model**							**−6.53**	**[−16.61; 3.56]**	**36.3%**
Heterogeneity: $I^2 = 0\%$, $\tau^2 = 0$, $p = 0.8789$									
**Random effects model**							**−5.88**	**[−11.14; −0.61]**	**100.0%**

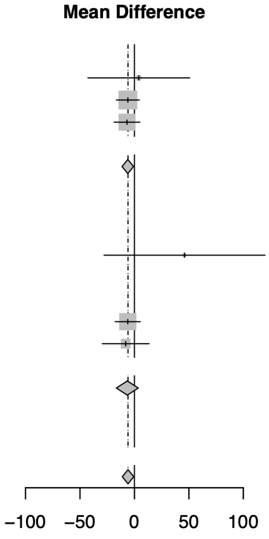

Heterogeneity: $I^2 = 0.0\%$, $\tau^2 < 0.0001$, $p = 0.8316$

Test for subgroup differences (random effects): $\chi^2_2 = 2.03$, df = 2 ($p = 0.3631$)

Supplementary figure 2. Subgroup analysis for the intraoperative blood loss outcome.

VAS preoperative axial pain

Study	Experimental Total	Experimental Mean	Experimental SD	Control Total	Control Mean	Control SD	MD	95%-CI	Weight
Lin, 2023	47	5.20	1.4000	43	5.30	1.5000	-0.10	[-0.70; 0.50]	21.0%
Siller, 2020 C	20	5.00	1.9000	20	5.00	1.9000	0.00	[-1.18; 1.18]	5.5%
Lin, 2022	23	6.30	1.2000	27	6.20	1.4000	0.10	[-0.62; 0.82]	14.6%
Yao, 2022	22	6.70	1.2000	25	6.50	1.1000	0.20	[-0.46; 0.86]	17.3%
Yao, 2021	21	5.90	0.8000	27	5.60	0.9000	0.30	[-0.18; 0.78]	32.6%
Siller, 2020 L	40	7.00	2.2000	40	6.00	2.0000	1.00	[0.08; 1.92]	8.9%
**Random effects model**	**173**			**182**			**0.22**	**[-0.12; 0.55]**	**100.0%**
**Prediction interval**								**[-0.15; 0.58]**	

Heterogeneity: I^2 = 0.0%, τ^2 = 0, p = 0.5223

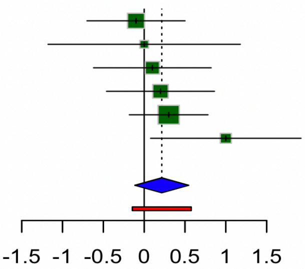

VAS postoperative axial pain

Study	Experimental Total	Experimental Mean	Experimental SD	Control Total	Control Mean	Control SD	MD	95%-CI	Weight
Siller, 2020 L	40	0.00	2.2000	40	2.00	1.7000	-2.00	[-2.86; -1.14]	16.9%
Yao, 2022	22	2.50	0.7000	25	3.00	0.8000	-0.50	[-0.93; -0.07]	21.3%
Yao, 2021	21	3.90	0.8000	27	4.30	0.8000	-0.40	[-0.86; 0.06]	21.1%
Siller, 2020 C	20	0.00	0.0000	20	0.00	0.0000	0.00		0.0%
Lin, 2022	23	1.30	0.9000	27	1.30	0.8000	0.00	[-0.48; 0.48]	20.9%
Lin, 2023	47	4.40	1.5000	43	4.20	1.4000	0.20	[-0.40; 0.80]	19.7%
**Random effects model**	**173**			**182**			**-0.49**	**[-1.50; 0.52]**	**100.0%**
**Prediction interval**								**[-2.69; 1.71]**	

Heterogeneity: I^2 = 79.9%, τ^2 = 0.5072, p = 0.0005

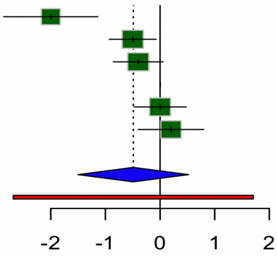

VAS preoperative radicular pain

Study	Experimental Total	Experimental Mean	Experimental SD	Control Total	Control Mean	Control SD	MD	95%-CI	Weight
Yao, 2022	22	5.10	1.3000	25	5.40	1.6000	-0.30	[-1.13; 0.53]	26.5%
Lin, 2023	47	6.40	1.7000	43	6.50	1.5000	-0.10	[-0.76; 0.56]	33.5%
Siller, 2020 C	20	5.00	1.9000	20	5.00	1.9000	0.00	[-1.18; 1.18]	16.7%
Siller, 2020 L	40	7.00	2.2000	40	6.00	2.0000	1.00	[0.08; 1.92]	23.3%
**Random effects model**	**129**			**128**			**0.12**	**[-0.79; 1.03]**	**100.0%**
**Prediction interval**								**[-1.35; 1.59]**	

Heterogeneity: I^2 = 39.2%, τ^2 = 0.1308, p = 0.1766

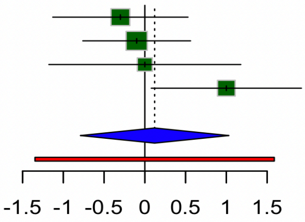

VAS postoperative radicular pain

Study	Experimental Total	Experimental Mean	Experimental SD	Control Total	Control Mean	Control SD	MD	95%-CI	Weight
Siller, 2020 L	40	0.00	1.7000	40	1.00	1.2000	-1.00	[-1.64; -0.36]	20.4%
Lin, 2023	47	3.90	1.5000	43	4.10	1.4000	-0.20	[-0.80; 0.40]	22.0%
Yao, 2022	22	2.10	0.6000	25	2.20	0.9000	-0.10	[-0.53; 0.33]	28.8%
Siller, 2020 C	20	0.00	0.7000	20	0.00	0.7000	0.00	[-0.43; 0.43]	28.8%
**Random effects model**	**129**			**128**			**-0.28**	**[-0.96; 0.41]**	**100.0%**
**Prediction interval**								**[-1.46; 0.90]**	

Heterogeneity: I^2 = 56.6%, τ^2 = 0.0960, p = 0.0749

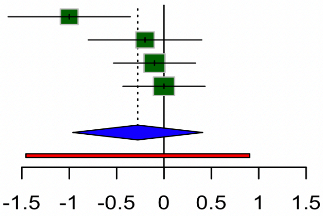

Supplementary figure 3. Subgroup analysis for the pain outcomes.

Supplementary table 1. Qualitative assessment of non-clinical parameters across 4 included studies. Red: inferiority of the exoscope as compared to microscope; grey: same performance of exoscope and microscope. Green: superiority of the exoscope as compared to microscope.

	Handling	Maneuvering	Comfort	Focus/ optic adjustment	Image quality	Depth	illumination	Didactics/team involvement	Glasses experience
Siller 2022H									
Lin 2022									
Yao, 2022									
Lin 2023									
